# Guillain-Barré Syndrome Following the Administration of Adenovirus Vector-Based COVID-19 Vaccine

**DOI:** 10.7759/cureus.42316

**Published:** 2023-07-23

**Authors:** Mardy L Lee, Juan Miguel P Bautista

**Affiliations:** 1 Neurology, Quirino Memorial Medical Center, Quezon City, PHL

**Keywords:** bilateral symmetrical lower extremity weakness, viral vector-based vaccine, philippines covid-19 vaccination program, guillain-barré syndrome, gbs, covid, adenovirus vector based vaccine, covid 19 vaccine complication

## Abstract

As countries worldwide deployed their respective coronavirus disease 2019 (COVID-19) vaccination programs to mitigate the risk of COVID-19 to their citizens, several side effects and complications from the use of the various types of COVID-19 vaccines were documented and are continued to be monitored to further study the safety and efficacy of these vaccines. One such complication, the Guillain-Barré syndrome (GBS), was reported in some individuals who received a COVID-19 adenovirus vector-based vaccine. In this particular report, we describe one such case. The patient had diarrhea and fever one day after vaccination, which was a triggering event. Seven days post vaccination, the patient had bilateral symmetrical weakness with absent deep tendon reflexes. However, causality between the vaccine administered and the suspected adverse reaction cannot be readily assumed. The benefits and risk profiles of each available vaccine should be assessed continuously for it to be improved and truly useful in this pandemic. Thorough post-vaccination surveillance, along with national reporting mechanisms are needed to help establish and confirm possible links between GBS and adenovirus vector-based COVID-19 vaccines. This link needs to be probed further in prospective studies and clinical trials.

## Introduction

Guillain-Barré Syndrome (GBS) associated with coronavirus disease 2019 (COVID-19) has been reported in various places of the world. The WHO Global Advisory Committee on Vaccine Safety (GACVS) reviewed cases of GBS following vaccination with adenovirus vector-based COVID-19 vaccines. It is for this reason that companies that produce adenovirus vector-based COVID-19 vaccines have alerted healthcare workers on the signs and symptoms of GBS. At the same time, a potential increase had been also observed following the administration of mRNA COVID-19 vaccines [1,2]. However, published epidemiological surveillance data are still lacking.

Earlier studies linking the two were recorded from the H1N1 influenza A vaccination cases in the late 1990s to early 2000s [3-6]. A meta-analysis of the 2009 H1N1 influenza A vaccine with data from six adverse event monitoring systems in the United States found that the vaccine was associated with a small increased risk of GBS, estimated to be 1.6 per one million people vaccinated [4]. Similarly, a 2009 cohort study on the H1N1 influenza A vaccine done in Quebec, Canada, found a small but significantly increased risk of GBS during the four-week and eight-week post-vaccination follow-ups at approximately two per one million doses administered [5]. In an analysis of 550 first-episode cases of GBS from 1995 to 2006 in a northern California healthcare database, a diagnosis of recurrent GBS was observed in six cases or equivalent to 1% [3]. There were no cases of recurrent GBS among the 107 patients who received influenza vaccination after the initial diagnosis of GBS, and none of the six individuals with recurrent GBS had any vaccine exposure in the two months prior to the onset of the second episode of GBS [3].

Hence, while there is a small risk of developing GBS associated with influenza vaccination, this is notably less than the overall health risk due to naturally occurring influenza. Also, the increased risk of GBS as a complication of influenza infection could be several times greater than the associated risk of GBS following influenza vaccination [6].

Moving on to 2021 with the widespread use of COVID-19 adenovirus vector-based vaccines, the Pandemic Response Accountability Committee (PRAC) looked into 108 cases of GBS reported worldwide as of June 30, 2021, with over 21 million people receiving various types of COVID-19 vaccines worldwide [7,8]. 

Moreover, the VigiBase analysis reported adverse drug reactions are associated with an increased observed-to-expected ratio of GBS, exceeding 2.0. Sputnik V adenoviral vaccine also reported cases of GBS after administration. After assessing the available data, there could be a possible causal relationship between the COVID-19 vaccine and GBS [9-11].

The Philippines’ COVID-19 vaccination program was deployed and rolled out in March 2021. There are currently eight vaccines that were given emergency use authorization in the country. These are by Moderna, Inc. (Massachusetts, United States), Pfizer/BioNTech (Pfizer Inc., New York, United States; BioNTech SE, Germany), Gamaleya Research Institute (Moscow, Russia), Janssen Pharmaceuticals (Beerse, Belgium), Oxford/AstraZeneca (Oxford University, Oxford, United Kingdom; AstraZeneca plc, Cambridge, United Kingdom), Sinovac Biotech Ltd (Beijing, China), Sinopharm or China National Pharmaceutical Group Corporation (Beijing, China), and Bharat Biotech (Hyderabad, India). The viral vector-based vaccines are Sputnik V (Gamaleya Research Institute), Johnson & Johnson vaccine (Janssen Pharmaceuticals), and Oxford/AstraZeneca [12,13].

## Case presentation

**Figure 1:**
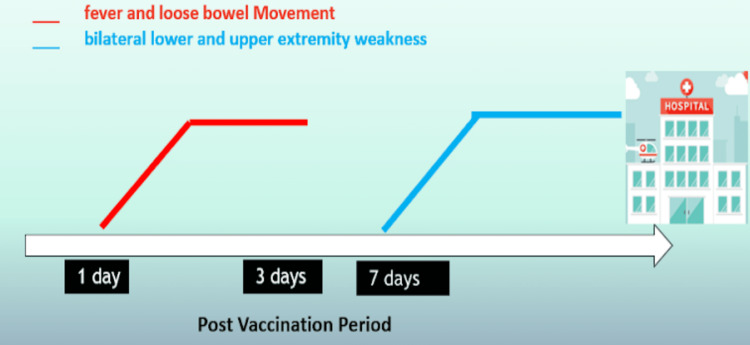
Post-vaccination signs and symptoms

A 32-year-old male presented with progressive weakness initially affecting the bilateral lower extremities and then spreading to the upper extremities starting seven days after receiving the first dose of the Oxford/AstraZeneca COVID-19 vaccine. Symptoms started the day after vaccination when he had four episodes per day of watery soft diarrhea with undocumented fever lasting for three days. The patient self-medicated with paracetamol and the symptoms eventually subsided. Seven days after vaccination, the patient started to have bilateral symmetrical lower extremity weakness associated with a tingling sensation in the toes and feet. This weakness eventually progressed affecting the bilateral upper extremities without sensory loss. No bowel or bladder symptoms, nor respiratory symptoms were reported at this time (Figure 1).

On examination, vital signs and general physical examination were normal. Neurological examination revealed bilateral symmetrical weakness of the upper and lower extremities with absent deep tendon reflexes and intact sensory function. No dysautonomia noted. Cerebellar, bulbar, and respiratory functions were normal, same with extraocular movements.

Reverse transcriptase-polymerase chain reaction (RT-PCR) swab test for severe acute respiratory syndrome coronavirus 2 (SARS-COV2) mRNA was negative as well as other routine laboratory tests for acute phase reactants. Blood tests pertaining to any systemic inflammation were also normal. A lumbar tap was done which revealed an opening pressure of 14 cmHg and a closing pressure of 10cmHg. CSF analysis showed an elevated CSF protein of 911 mg/L with no cells on the differential count. The electromyography (EMG) and nerve conduction velocity (NCV)study showed an acute, severe, diffuse, sensorimotor polyradiculoneuropathy of the upper and lower extremities which was axonal in nature and may be seen in acute motor sensory axonal neuropathy (AMSAN) variants of GBS. 

After five days of intravenous immunoglobulin (IVIG), the patient improved and was able to ambulate without sensorimotor deficits.

## Discussion

GBS is a neurological disorder involving acute polyneuropathy that is thought to result from molecular mimicry where the immune response to a preceding infection or other event cross-reacts with peripheral nerve components [10]. Although rare, it is an adverse event of interest for all COVID-19 vaccines [14]. Adenovirus-based vaccines are made from a modification of adenovirus to produce the SARS CoV-2 spike protein [8,13,14].

The demyelinating and axonal forms are borne from the immune response that can be directed toward the myelin or the axon of peripheral nerves, respectively [14]. Affected individuals have a history of mild respiratory or gastrointestinal infection preceding the neuropathic symptoms by at least one to three weeks. Symptoms include paresthesia and slight numbness in the toes and fingers, weakness involving limb muscles with signs of reduced or absent deep tendon reflexes, or facial diplegia [15].

In a report by Allen et al., the post-vaccination host antibodies generated may cross-react with proteins present in the peripheral myelin resulting in the neurological syndrome typical of GBS [15]. These antibodies can be directly reactive to SARS-CoV-2 spike protein, but may also involve a less specific immune response, e.g., to components of the adenovirus vector. Evidence shows that the SARS-CoV-2 spike protein can bind to sialic acid-containing glycoprotein and gangliosides on cell surfaces, which also increases its viral transmissibility [15]. The pathogenesis of COVID-19 infection or immunization-associated GBS may result from the antibody cross-reactivity between the SARS-CoV-2 spike protein and peripheral nerve glycolipids. As seen in other autoimmune neurologic disorders, COVID-19-associated GBS may be influenced by a host’s specific genetic makeup, such as the human leukocyte antigen haplotype profile [15].

Maramathom et.al. surmised a Naranjo adverse drug reaction of 3, which could indicate a possible association between vaccination and GBS [16]. In their study, all patients progressed to areflexia quadriplegia, and six of the seven cases required mechanical ventilation for respiratory failure [16]. Allen et. al. reported four cases of GBS following vaccination with the AstraZeneca COVID-19 vaccine [15]. However, the reported presentation of these four cases was facial diplegia with paresthesia. All cases happened within 10 days after vaccination [15].

Post-vaccination phenomenon is rarely discussed in the literature. However, adenovirus-based vaccine literature has provided warnings on the rise of GBS following vaccination [1,7,8,15].

The Food and Drug Administration (FDA) of the Philippines said no case of GBS has been reported or recorded as of April 2021, according to a summary of the FDA report of suspected adverse drug reactions to COVID-19 vaccines [14]. The agency documented that most of the adverse reactions include the following: body pain, chills, fatigue, fever, headache, nausea, and pain on the injection site, usually appearing on the first or second-day post vaccination and lasting about two to three days after symptom onset [12]. These adverse reactions are mostly well tolerated by most patients, while a minority of patients may experience greater discomfort [12].

In the current case report, the patient had gastrointestinal symptoms of diarrhea and fever one day after vaccination which was a triggering event. He had a bilateral symmetrical weakness with absent deep tendon reflexes seven days post vaccination. The CSF analysis showed an elevation of CSF protein and normal CSF cell count. Although the patient had neurological symptoms of GBS following COVID-19 vaccination, the link between the two cannot be assumed.

Vaccine adverse reactions are considered as serious when they result in patient hospitalization, prolonged hospitalization, or when there is significant disability or incapacity [12], as was reported in this case report.

## Conclusions

With the possibility of having an acute inflammatory demyelinating polyneuropathy after administration of a vector-based vaccine for COVID-19, healthcare workers who administer vaccination should be more aware and active in advising patients to watch out for signs and symptoms of GBS for early detection, diagnosis, and treatment. The benefits and risk profiles of each available vaccine should be assessed continuously for it to be improved and truly useful in this pandemic. Monitoring and reporting adverse events are necessary for the safety assessment of different COVID-19 vaccines.

At this point, causality between the vaccine administered and the suspected adverse reaction cannot be readily assumed. This data will add to the existing reports on GBS associated with COVID-19 vaccines. The link between GBS and the adenovirus vector-based COVID-19 vaccines needs to be probed further in prospective studies and clinical trials.
